# Oxidative stress and mitochondrial uncoupling protein 2 expression in hepatic steatosis induced by exposure to xenobiotic DDE and high fat diet in male Wistar rats

**DOI:** 10.1371/journal.pone.0215955

**Published:** 2019-04-25

**Authors:** Vincenzo Migliaccio, Rosaria Scudiero, Raffaella Sica, Lillà Lionetti, Rosalba Putti

**Affiliations:** 1 Department of Biology, University of Naples, Federico II, Naples, Italy; 2 Department of Chemistry and Biology “Adolfo Zambelli”, University of Salerno, Fisciano, Italy; National Institutes of Health, UNITED STATES

## Abstract

Oxidative stress plays a key role in steatohepatitis induced by both xenobiotic agents and high fat diet (HFD). The present study aimed to evaluate hepatic oxidative stress and anti-oxidant systems response in rats exposed to HFD and/or non-toxic dose of dichlorodiphenyldichloroethylene (DDE), the first metabolite of dichlorodiphenyltrichloroethane. Groups of 8 rats were so treated for 4 weeks: 1- standard diet (N group); 2- standard diet plus DDE (10 mg/kg b.w.) (N+DDE group); 3- HFD (D group); 4- HFD plus DDE (D+DDE group). Oxidative stress was analyzed by determining malondialdehyde as lipid peroxidation product, while the anti-oxidant systems were evaluating by measuring the levels of the principal cytosolic and mitochondrial antioxidant proteins and enzymes, namely superoxide dismutase 1 and 2 (SOD1, SOD2), glutathione peroxidase 1 (GPx1) and uncoupling protein 2 (UCP2) involved in the control of hepatic reactive oxygens species (ROS) accumulation. The results showed malondialdehyde accumulation in livers of all groups, confirming the pro-oxidant effects of both HFD and DDE, but with a greater effect of DDE in absence of HFD. In addition, we found different levels of the analyzed anti-oxidant systems in the different groups. DDE mainly induced UCP2 and SOD2, while HFD mainly induced GPx1. Noteworthy, in the condition of simultaneous exposure to DDE and HFD, the anti-oxidant response was more similar to the one induced by HFD than to the response induced by DDE. Present findings confirmed that both HFD and xenobiotic exposure induced hepatic oxidative stress and showed that the anti-oxidant defense response was not the same in the diverse groups, suggesting that UCP2 induction could be an adaptive response to limit excessive ROS damage, mainly in condition of xenobiotic exposure.

## Introduction

The liver is the main organ involved in xenobiotic detoxification as well as in dietary lipid metabolism, and hepatic steatosis is the most common pathologic liver responses to both high fat diet (HFD) and chemical exposures [1]. The metabolism disrupting chemical (MDC) hypothesis suggested by Heindel et al., (2017) [2] postulates that environmental chemicals have the ability to promote metabolic changes that can result in obesity, diabetes and/or fatty liver disease. MDC hypothesis provides a framework for the integration of different aetiology of steatohepatitis: alcoholic, non-alcoholic and toxicant-associated steatohepatitis (ASH, NASH, and TASH, respectively). A common mechanism for the etiologically different liver diseases may be found in inflammation and oxidative stress. It is well known that HFD induced hepatic mitochondrial dysfunction and oxidative stress [3,4,5,6]. On the other hand, liver xenobiotic metabolism may increase oxidative stress [7]. Little is known on the effect of simultaneous exposure to xenobiotics and HFD on liver oxidative stress and metabolic disorders. Under physiological conditions, low levels of ROS are essential in many biochemical processes, including intracellular signalling, defence against microorganisms, and cell function. On the contrary, the excessive production of ROS modifies the balance between the oxidants / prooxidants and antioxidants agents, leading to lipid peroxidation and depleting the antioxidant cellular reserves (both enzymatic and non-enzymatic), causing tissue injury and, in many cases, apoptosis. Among xenobiotic agents, the pesticide dichlorodiphenylethylene (DDE) is the most persistent metabolite of the insecticide dichlorodiphenyltrichloroethane (DDT) and causes hepatoxicity, nephrotoxicity and hormonal disorders [8,9]. Moreover, it produces mitochondrial dysfunction [10] and oxidative stress in different organisms, such as marine species [11], terrestrial vertebrates [12] and cell culture [13]. Today, DDT utilization against the principal disease vectors is restricted to equatorial countries, where malaria is still endemic [14]. Nevertheless, residues of DDT and DDE are still observed in soils of many occidental countries, and in mother’s milk [15], in maternal blood serum [16] and in grapes [17]. DDT was listed by the Convention on Persistent Organic Pollutants in the “Dirty Dozen” substances in 2001. However, apart from the tropical countries where DDT is still currently used, several other countries are considering the possibility to reintroduce it [18]. In literature it was reported that DDE toxicity is due mostly to ROS production [19]. In the hepatocytes, the first line of defense from free radicals is represented by the superoxide dismutase (SODs) that catalyze the dismutation of superoxide in H_2_O_2_ and oxygen. Three isoforms of SODs have been identified, each expression of a different gene and with distinct subcellular localizations. Cu/ZnSOD (SOD1) is a cytosolic enzyme, MnSOD (SOD2) has a mitochondrial localization, and EC-SOD (SOD3) is localized in the extracellular matrix, being secreted from cells [20]. SOD1 is constitutively expressed, but can be induced by redox-active metals, superoxide, and xenobiotics. SOD2 is the most inducible form, raising its levels up to10-fold in presence of drugs and cytokines. Defects in SOD2 expression cause oxidative damage in liver, while the overexpression generally plays a protective role [21]. SOD3 does not have a significant role in superoxide detoxification in hepatocytes [22]. Another class of intracellular antioxidant enzyme are known as glutathione peroxidase (GPx). GPx are tetrameric enzymes containing a seleno-cysteine in their active site [23]. These enzymes occur in different isoforms (eight in humans), all able to degrade hydroperoxides, alkyl peroxide, and fatty acid hydroperoxides to lipid alcohols and oxygen. In particular, GPx1 seems to be the major isoform that converts hydrogen peroxide to water and oxygen and catalyzes the reduction of peroxide radicals to alcohols and oxygen; mainly cytosolic, a small fraction is also present within the mitochondrial matrix [24]. GPx1 exerts its action via oxidation of reduced GSH into its disulfide form [25]. It is known that the mitochondrial respiratory chain is the major site of intracellular ROS generation and, at the same time, an important target for the ROS damaging effects. The superoxide anion produced in the matrix side of inner mitochondrial membrane has been proposed to activate the uncoupling proteins (UCPs) that in turn might reduce the generation of further superoxide anions [26]. UCPs are a family of mitochondrial proteins formed by six trans-membrane segments into the phospholipid matrix of the inner mitochondrial membrane [27], present in animal and plant in five isoforms, from UCP1 to UCP5 [28]. UCPs isoforms are expressed in different tissues and may have different functions. The isoform1 is present in brown adipose tissue (BAT), isoform 2 is expressed almost ubiquitously, isoform 3 in BAT and in skeletal muscle, isoforms 4 and 5 are present predominantly in the central nervous system [29]. Moreover, in mitochondria, UCPs functional structure is constituted by a dimer stabilized with a disulfide bridge between the cysteines present in the hydrophilic C-terminal segment [30]. These proteins are mitochondrial anion carriers [31] whose function was initially associated to uncoupling respiration from ATP synthesis performed by UCP1, the first isoform to be discovered by Nicholls and coworkers (1978) [32], that determines releasing of heat from the oxidation of substrates in brown adipocytes. Besides the adaptive thermogenesis [33], UCPs may regulate a lot of biological processes, such as the ATP synthesis and all the mechanisms directly or indirectly linked to ATP utilization, for example the inhibition of insulin secretion by UCP2 from the pancreatic beta cells [34]. The ubiquitously UCP2 is described as mitochondrial scavenger of ROS produced by mitochondria [35, 36]. The antioxidant effect of UCP2 has been reported by in vitro and in vivo studies using UCP2 overexpression, genetic ablation and pharmacological inhibition [37, 38, 39]. Moreover, different research works suggest that UCP2 could be involved in lipid metabolism: it could stimulate fatty acids oxidation and/or prevent the oxidative damage due to high lipid levels [40]. In many tissues it has been found a modulation of UCP2 expression, with both a basal and stimulated synthesis of the protein [41]. In liver, under physiological conditions, UCP2 is essentially localized in the immunocompetent cells [42], while in conditions of oxidative stress with mitochondrial ROS accumulation, the protein is up-regulated and expressed in hepatocytes [43], suggesting that UCP2 plays an important role as a negative regulator of mitochondrial ROS production [35]. To clarify if DDE toxicity on liver is due predominantly to the oxidative stress caused by this pesticide, we administered male Wistar rats with a daily dose of DDE comparable to human daily absorption or blood concentration [44, 45]. We also compared the effect of DDE to the effect of HFD treatment on hepatic oxidative stress onset. Finally, we analyzed the effect of the simultaneous exposure to both HFD and DDE on the same markers of oxidative stress. To this end, we determined the activation of the antioxidant enzymatic systems (SOD1-SOD2) and GPx1 response and we tested the hypothesis of mitochondrial uncoupling involvement to prevent ROS production in terms of UCP2 gene expression and protein synthesis in rat livers. Our findings showed, together with the activation of the antioxidant enzymatic systems, a difference in UCP2 modulation according to the treatments used, with the highest induction of UCP2 in the hepatocytes of DDE-treated animals. From these data we assumed that UCP2 plays a protective role to limit cell damage and liver injury mitigating mitochondrial ROS production with an increasing functional impact at increasing levels of oxidative stress.

## Materials and methods

### Ethics statement

This study was performed in accordance with recommendations in the EU Directive p2010/63/ for the Care and Use of Laboratory Animals. The protocol was approved by the Committee on the Ethics of Animal Experiments of the University of Naples Federico II (Permit Number: 2012/0024690).

### Experimental model

As experimental model 32 male *Wistar* rats (Charles River Italia, Calco, Como, Italy) were used. Since the environmental exposure to DDE is in general the result of the introduction of low doses through the food, in this study we decided to administer DDE at low doses (10 mg/kg b.w.) orally. It has been reported that the oral administration of this DDE dose does not influenced physical development, sexual maturation, and serum metabolic parameters in male pubertal or older rats when is administered for 6 or 4 weeks, respectively [46]. At the start of the study, the rats were divided into the following four groups (including eight rats each) with a similar mean body weight (approximately 300 g) and with the body weights normally distributed within each group: N group (standard control diet PF1915 from Mucedola, Milano, Italy, 0.6% fat J/J, 15.47 KJ/g,); N+ DDE group (standard control diet, +10mg/kg b.w. of DDE); D group (high fat diet, PF1916 from Mucedola, Milano, Italy, 45% fat J/J, 19.88KJ/g) and D+DDE group (high fat diet, +10mg/kg b.w. of DDE). All rats were housed individually, acclimatized in a temperature-controlled room (24° C) and subjected to a circadian light-dark cycle (12 hours light / 12 hours dark). At the end of the experimental period of 4 weeks, the rats were anesthetized by an intraperitoneal injection of Zoletil (40 mg/Kg body weight) and euthanized by decapitation. Retroperitoneal and epididymal white adipose tissue (WAT) pads and liver were immediately removed and weighted. Liver slices were either immediately processed for morphological analysis and mitochondria isolation, or frozen in liquid nitrogen and stored at -80°C for later processing.

### Determination of serum parameters

Serum levels of triglycerides and cholesterol levels were determined by colorimetric assay kit (Cayman Chemical Company, No.10010303) and fluorometric assay kit (Cayman Chemical Company, No. 10007640), respectively. Serum alanine aminotransferase (ALT) and aspartate aminotransferase (AST) levels were determined by standard procedures (colorimetric kit by Cayman Chemical Company (No. 700260) for ALT and ELISA kit by Fine Test Biotech Company (No. ER0748) for AST).

### Hepatic lipid content

Hepatic lipid content was determined gravimetrically after extraction in chloroform–methanol and evaporation to constant weight with a rotating evaporator (Heidolh, Germany) according to the method described by Folch et al., (1956) [47].

### Morphological analysis

Liver slices were washed in cold ice NaCl 0.9% for few seconds and fixed in Bouin’fluid for 12 hours. Then, each liver fragment was dehydrated, embedded in paraplast and cut at 5 μm. The histological sections were processed by Hematoxylin & Eosin stain.

### Oxidative stress parameters

#### Thiobarbituric acid reactive substances–TBARS assay

To evaluate the effect of the treatments on the lipid peroxidation was used TBARS assay kit, (Cayman Chemical Company,No.10009055). 25 mg of fresh liver tissue for each animal were washed in cold ice PBS (1.4 mM KH_2_PO_4_, 8 mM Na_2_HPO_4_, 140 mM NaCl, 2.7 mM KCl, pH 7.4) and homogenated in 250μL of RIPA Buffer (150mM NaCl, 50mM Tris pH 7.4, 1% NP-40, 0.5% sodium deoxycholate, 0.1% SDS) plus a cocktail of protease inhibitors (Sigma Aldrich Chemicals). The homogenate was centrifuged at 1600xg for 15 minutes and the resulting supernatant was used for the analysis. All solutions were prepared as indicated by the manufacturer. The Malondialdehyde-Thiobarbituric acid adducts (MDA-TBA) in the samples were measured spectrophotometrically at 532nm. An additional spectrophotometric measure was made at 600nm to detract the non-specific TBA adducts with other aldehydes formed during the peroxidation events. The amount of MDA in each sample, expressed as nmol of MDA per mg of proteins, was calculated by using MDA standard curve and the MDA molar extinction coefficient. The results were reported as fold change of MDA content in all treated groups vs. N group.

#### Hepatic oxidized glutathione (GSSG) and antioxidant enzymes levels

The hepatic levels of GSSG were obtained by using a colorimetric kit (Cayman Chemical Company, No. 703002).

Total SODs (Cu/Zn-SOD and Mn-SOD) activity and specific Mn-SOD (SOD2) activity were monitored on total liver homogenate by using a colorimetric kit (Cayman Chemical Company, No.706002). For SOD2 activity detection, potassium cyanide 3mM was added to inhibit SOD1 and SOD3 according to the manufacturer.

Total GPx activity was obtained by using a colorimetric kit (Cayman Chemical Company, No. 703002).

### Preparation of mitochondrial fraction

Mitochondria isolation was performed as described by Lionetti et al. (2004) [48]. Liver fragments were washed and homogenized in 220mM Mannitol, 70mM sucrose, 20mM HEPES, 1mM EDTA and 0.1% fatty acid-free BSA, pH 7.4 (1:10, w/v) with a Potter Elvehjem homogenizer (Heidolph, Kelheim, Germany) set to 500 rpm (4 strokes/min). The homogenate was filtrated and centrifuged at 1000g for 10 minutes. The pellet was discarded and the supernatant, containing mitochondria, was rapidly centrifuged at 3000g for 15 min. The final pellet was made of a subcellular fraction essentially constituted by mitochondria [49]. Mitochondrial suspension was washed for three times and resuspended in 80mM LiCl, 50mM HEPES, 5mM Tris phosphate buffer pH 7.4, 1mM EGTA, and 0.1% fatty acid-free BSA, containing a cocktail of protease inhibitors (Sigma Aldrich Chemicals). Finally, mitochondrial suspension was lysed (1:1 v/v) in RIPA buffer solution (150 mM NaCl, 50 mM Tris, 1% NP-40, 0.25% sodium deoxycholate, 0.1% SDS, pH 7.4) by 10 steps in insulin syringe with thin needle. The resulting mitochondrial homogenate was centrifuged at 12,000g for 10 minutes. The pellet was discarded, and the supernatant was used for analysis.

### Mitochondrial fatty acid oxidation rate and carnitine palmitoyl-transferase system (CPT) activity assay

The rate of mitochondrial fatty acid oxidation was assessed in the presence of palmitoyl-l-carnitine (40 μM) by using a Clark-type electrode as previously reported [5]. CPT system (CPT1 plus CPT2) activity was measured spectrophotometrically (at 412 nm) as described by Alexson et al., (1988) [50].

### Preparation of total homogenate

Total proteins from liver were obtained using RIPA buffer solution (150 mM NaCl, 50 mM Tris, 1% NP-40, 0.25% sodium deoxycholate, 0.1% SDS, pH 7.4) containing a cocktail of protease inhibitors (Sigma Aldrich Chemicals). Liver fragments were homogenates using a polytron (KINEMATICA Polytron Model PT10-35 GT/PT 3100D Homogenizer, Fisher Scientific) and centrifuged at 12,000g for 10 minutes. The pellet was discarded, and the supernatant was used for analysis.

### RNA isolation and cDNA synthesis

Total RNA was extracted from liver fragments according to the Tri-Reagent (Sigma-Aldrich) protocol. The quality of each total RNA was checked by electrophoresis on 2% agarose gel stained with ethidium bromide and measuring the optical density at 260/280 nm. A ratio of 1.8–2.0 was accepted for further reverse transcription. QuantiTect Reverse Transcription Kit (Qiagen) was used for the removal of genomic DNA contamination and for the subsequent cDNA synthesis. Approximately 1μg of total RNA was used, according to the kit’s protocol.

### UCP2 cDNA sequencing

The cDNA corresponding to a fragment of the UCP2 coding sequence was cloned starting from reverse-transcribed RNA from liver of a control rat (N group). Conventional PCR reactions were carried out on 2 μl of first-strand cDNA, using the forward primer 5’-AGCAGTTCTACACCAAGGGC-3’ and the reverse primer 5’-AGAGGTCCCTTTCCAGAGGC-3’, designed on the exon junction 532/533 (reverse primer) on template NM019354.2. Primer sequences were designed using Primer Express software (Applied Biosystems). The analysis retrieved a single cDNA fragment of 230 bp that was purified and inserted into the pSC-A vector using the Strataclone PCR Cloning Kit (Agilent Technologies) according to the manufacturer’s instructions. The cDNA fragment was sequenced using automated methods on an ABI PRISM Genetic Analyzer (PE Biosystems), showing 100% homology with the *Rattus norvegicus* UCP2 gene.

### Quantitative real-time PCR analysis

UCP2 mRNA was quantified with qReal-Time PCR. The analyses were carried out on an Applied Biosystem 7500 Real-Time PCR System, using the Power SYBR Green PCR Master Mix (Life Technologies), following the procedures recommended by the manufacturer. Each amplification mixture of 20 μl final volume contained 12 μl of real-time PCR Master Mix, 1 μl each of UCP2 forward and reverse primers described above, 2 μl of cDNA diluted 1:1 and 4 μl of nuclease free water. Amplifications were performed with an initial step at 95°C for 1 minute, followed by 40 cycles at 95°C for 15 s and 60°C for 40 s. A melting curve analysis of PCR products was performed from 60°C to 95°C in order to ensure gene specific amplification. For internal standard control, the expression of β-actin gene was also quantified; β-actin primers were designed on the exon junction 75/76 (forward primer) on template NM031144.2 [51]. Changes in the UCP2 gene expression in the different samples were obtained in according to the standard 2^−ΔΔCt^ method described by [52].

### *In Situ* hybridization

After slide preparation, as previously reported, the sections were incubated with Proteinase K 10μg/ml at 37°C (5 min.) in 1 M Tris/HCl pH 7.0, 0.5 M EDTA pH 7.2, to eliminate the endogenous DNAse and RNAse activity. Sections were then treated for 60 min. with a pre-hybridation mix solution containing 50% formamide, 1X Denhardt’s, 100 μg/ml salmon sperm and 100μg/ml t-RNA in DEPC water at 55°C. Hybridization was carried out at 42°C overnight with the UCP2 digoxigenin-labeled probe in a humid chamber. Slides were then washed three time with decreasing sodium chloride/sodium citrate solutions (2X SSC, 1X SSC, 0.5X SSC) containing 50% formamide and blocked in 2% Blocking solution in Maleic acid for 30 min. at room temperature. Then, the sections were incubated overnight at 4°C with an alkaline phosphatase-conjugated anti-Dig antibody (Roche Diagnostics) diluted 1:2500 in 2% Blocking solution. Sections were repeatedly washed in 100mM TBS, 1X-Tween-20, and the reaction was revelated using BM-purple (Roche, N and N+DDE groups) and BCIP-NBT (Roche, D and D+DDE groups). Finally, the reaction was blocked in PBS containing 1mM EDTA and the slides mounted with aqueous mounting medium and coverslips. Dig-labelled UCP2 cDNA probe was generated by PCR using the DIG High Prime DNA labelling and detection starter kit I (Roche). In the negative control, the hybridization solution did not contain the labelled probe.

### UCP2 immunolocalization

Immunohistochemical reactions were performed using Novolink Polymer Detection Systems (RE7280-CE, Leica Biosystems). After antigen retrieval and quenching of endogenous peroxidase, sections were incubated overnight at 4°C with UCP-2 (goat-polyclonal antibody C-20, sc-6525), diluted 1:200 in PBS, and CD68 (mouse-monoclonal antibody, sc-59103) diluted in PBS 1:100, to detect Kupffer cells. The sections were then incubated in Novolink polymer 1h at RT, and immunostaining was performed using 3,3′-diaminobenzidine (DAB) as chromogen. The sections were counterstained with hematoxylin and mounted with a coverslip. To test the specificity of the reagents, the following controls were performed: (a) omission of the primary antiserum and incubation of the sections with either non-immune serum (1:10 in PBS) or 1% bovine serum albumin (BSA; Sigma); (b) absorption of the optimally diluted primary antiserum with its specific peptide (10 nmol/ml of optimally diluted antiserum) for 24 h at 4°C. When the specific peptide was used, the staining was abolished. Images of sections were acquired using a Zeiss Axioskop microscope fitted with a TV camera.

### Western blotting

Western blotting analyses were performed on total liver homogenate to verify the amount of SOD1 and GPx1, and on mitochondrial protein extract to measure UCP2 and SOD2 levels in mitochondria. As loading control, endogenous specific protein markers were used: COXIV, as mitochondrial control, and β-actin for total protein extract. Western blot for UCP2 is often a problem for the lack of specificity of commercial UCPs antibodies against the different UCP isoforms. For this reason, several commercial UCP2 antibodies were validated and the classical western blot protocol was slightly modified. Indeed, prior to the SDS-PAGE, mitochondrial protein extract was pre-treated with iodoacetamide (Sigma Chemicals) to alkylate the cysteines, as described Sechi & Chait, (1989) [53]. This procedure avoided the formation of a proteic band of 70 KDa, likely due to the UCP2 dimerization, and allowed us to obtain only the predicted band at about 34 KDa, corresponding to the molecular mass of the UCP2 monomer. Among tested antibodies, the goat-polyclonal Antibody (C-20: sc-6525) was chosen for the subsequent analyses because retrieved a single UCP2 band at about 34KDa in both liver and spleen, used as positive control tissue. After mitochondrial and total homogenate preparation, the protein content was determined by the method of Hartree [54] using BSA as the protein standard. For both mitochondrial and total homogenates, 80μg of denatured proteins were electrophoresed in a 13% SDS–polyacrylamide gel as described by Laemmli [55], together with a pre-stained protein marker (ColorBurstElectrophoresis Marker m.w. 8–220 KDa, Sigma Aldrich). After the run, the proteins were transferred onto nitrocellulose membranes (Immobilon-P, Millipore, Switzerland) at 350mA for 60 minutes. The membranes were blocked in blocking buffer solution (1×TBS/ 1% Tween-20, 5% milk) for 60 minutes at room temperature and incubated overnight at 4°C in milk/TBS-Tween buffer (1×TBS/1% Tween-20, 2% milk) with the following antibodies: UCP-2, goat-polyclonal antibody (C-20): sc-6525, 1:200; UCP-1, rabbit-polyclonal antibody, AB1426, 1:1000; UCP-3, rabbit-polyclonal antibody, AB3477, 1:1000; SOD2, rabbit polyclonal antibody, Thermo Scientific PA5-30604, 1:500; GPx-1, rabbit polyclonal antibody, ThermoFisher scientific PA5-26323, 1:1000; SOD-1, rabbit polyclonal antibody, Thermo Scientific PA5-27240, 1:500; COX-IV, mouse-monoclonal antibody: sc-376731, 1:200; CYP450 2B1+2B2, mouse monoclonal antibody, AB22721, 1:1000; β-Actin, mouse monoclonal antibody: sc-70319, 1:200; Vinculin, mouse monoclonal antibody, sc-25336,1:200. Membranes were washed 4×15 min. with TBS-Tween solution and incubated for 1h at room temperature with a secondary antibody labeled with horseradish peroxidase, diluted in milk/TBS-Tween buffer (1×TBS/1% Tween-20, 5% milk). The secondary antibodies used were: Anti-mouse: Santa Cruz Biotechnology, goat-anti mouse, IgG-HRP: SC-2005, 1:5000; Anti-rabbit: Santa Cruz Biotechnology, donkey-anti rabbit, IgG-HRP: SC-2313, 1:5000; Anti-goat: Santa Cruz Biotechnology, donkey-anti goat, IgG-HRP: SC-2020, 1:5000. Membranes were washed 4×15 min with TBS-Tween solution and revealed with a chemiluminescent method, using Luminol solution (final concentration 2.5 mM) in presence of cumaric acids (final concentration 0.4 mM) and H_2_O_2_ (final concentration 100mM). The bands obtained were quantified using C-DiGit Chemiluminescent Western Blot Scanner (LI-COR).

### Statistical analysis

Data analysis was performed by calculating the mean of the values for each individual group ± standard deviation and shown, graphically, as fold changes of the treated groups vs. N. Statistical analyses were carried out with Graph pad software. Differences between values obtained to control and treated groups were analyzed by one-way analysis of variance (ANOVA) followed by Bonferroni post-test. The differences were considered significant when P value was inferior to 0.05.

## Results

### Body weight gain and serum metabolite levels

We first analysed whether the different treatments (HFD, DDE or HFD+DDE) differentially affected obesity development (body weight gain and weights of visceral fat pads), and serum levels of metabolites related to lipid metabolism (triglycerides and cholesterol levels) and hepatic injury (ALT and AST).

As regard obesity development, a significant increase in body weight gain was found in both D (+39%) and D+DDE group (+36%) vs. N group, whereas no significant change was found in N+DDE group compared to N (Table 1). In accordance, the weights of epididymal and retroperitoneal WAT pads were increased in D (+44% and +64%, respectively) and D+DDE (+60% and +75%, respectively) vs. N group, whereas no changes were found between N and N+DDE (Table 1).

**Table 1:** Body weight gain, serum metabolite parameters and hepatic lipid content.

	N	D	D+DDE	N+DDE
** Body weight gain (g)**	88.9±7.5	124.2±22.0 ***	121.2±19.8 •••	73.1±4.7
**WAT weight**				
** Epididymal WAT (g)**	9.5±1.4	13.7±1.2 ***	15.2±1.1 •••	10.6±1.0
** Retroperitoneal WAT (g)**	9.0±1.1	13.9±1.8 ***	15.8±1.2 #/•••	10.5±0.9
**Hepatic parameters**				
** Liver weight (g)**	12.8±0.5	12.9±0.6	14.0±0.9	13.3±1.0
** Lipid content (mg/g)**	4.2±0.2	7.3±0.8 ***	5.8±1.4 # #/••	4.3±0.3
**Serum parameters**				
** Triglycerides levels (mg/dL)**	139.9±8.6	237.6±3.0 ***	193.1±7.1 # # # •••	135.9±5.4 # # # ***
** Cholesterol levels (mg/dL)**	123.4±6.4	184.7±20.9 *	162.2±12.2 •	124.4±7.7 #
** ALT activity (U/mL)**	52.7±11.7	150.1±24.7 ***	133.5±7.4	152.0±11.5 ***
** AST content (ng/mL)**	1.81±0.46	6.15±1.61 ***	5.76±1.87	6.93±1.98 ***

WAT (white adipose tissue); ALT (Alanine Transaminase); AST (Aspartate Transaminase)

Data are reported as means±ES of 8 different rats for each group.

* p<0.05 compared vs. N

# p<0.05 compared vs. D

• p<0.05 vs. N+DDE

*** p<0.001 compared vs. N

### p<0.001 compared vs. D

••• p<0.001 compared vs. N+DDE.

N = rats fed with normal diet; D = rats fed with HFD; D+DDE = rats fed with HFD + 10mg/kg b.w. of DDE; N+DDE = rats fed with normal diet + 10mg/kg b.w. of DDE.

As regard serum metabolite parameters linked to body lipid metabolism, no changes in triglycerides levels were found between N and N+DDE groups, whereas significant triglycerides level increases were found in both high-fat fed rats (+70% and +39% in D and D+DDE, respectively) compared to control group (Table 1). Noteworthy, D+DDE group exhibits lower triglycerides levels (-30%) vs. D rats (Table 1).

Moreover, no changes were found in cholesterol level between N and N+DDE group, whereas increased cholesterol levels were found in both D (+50%) and D+DDE (+31%) vs. N group (Table 1).

The analyses of ALT activity and AST content were used to evaluate the amount of liver damage produced by the treatments. Increases in ALT activity were found in all the treated groups (~3-fold in D and N+DDE and 2.5-fold in D+DDE) vs. N group (Table 1). No significant change was detected between D and D+DDE. In accordance, AST serum content increased in treated groups (3.4-fold in D, 3.2-fold in D+DDE and 3.8-fold in N+DDE) vs. N group. No significant changes were found between D and D+DDE (Table 1).

### Hepatic morphological alteration, lipid content and oxidation

To investigate hepatic lipid accumulation, we used both histologic observations and lipid extraction according to the method of Folch [47]. No significant changes in liver weight were found among the experimental groups, whereas hepatic lipid content was higher in D (+74%) and D+DDE (+37%) compared to N group. Hepatic lipid content in N+DDE group was similar to that found in N group (Table 1).

**Fig 1:**
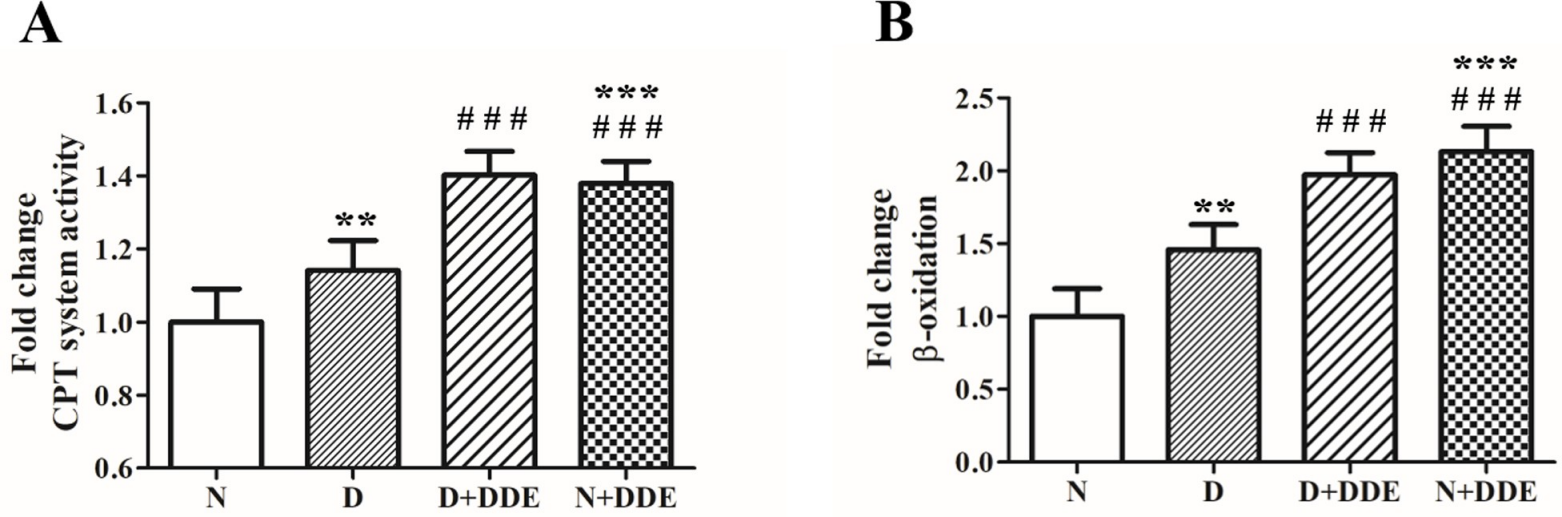
Hepatic mitochondrial lipid utilization. Fatty acid utilization capacity was tested measuring both CPT system activity (A) and β-oxidation rate (B). Data are reported as means±ES of 8 different rats for each group. Significant of differences is shown: ** *p<0*.*01* vs. N; *** *p<0*.*001* vs. N; ## *p<0*.*01* vs. D; ### *p<0*.*001* vs. D.

Moreover, mitochondrial fatty acid utilization was analyzed. A significant fold increase of CPT system activity was found in both HFD treated groups: 1.15-fold increase in D vs. N and ~1.20-fold increase in D+DDE vs. D. Noteworthy, CPT system activity also increased in N+DDE vs. N (~1.40-fold) and D (~1.20-fold), (Fig 1A). A similar trend was observed for mitochondrial fatty acid β-oxidation. Indeed, significant fold increase was found in D vs. N (~1.46-fold) and in D+DDE vs D (~1.35-fold). Fatty acid β-oxidation significantly increased also in N+DDE group vs. N (~2.00-fold) and D (~1.46-fold), similarly to the tendency found in D+DDE group (Fig 1B).

**Fig 2:**
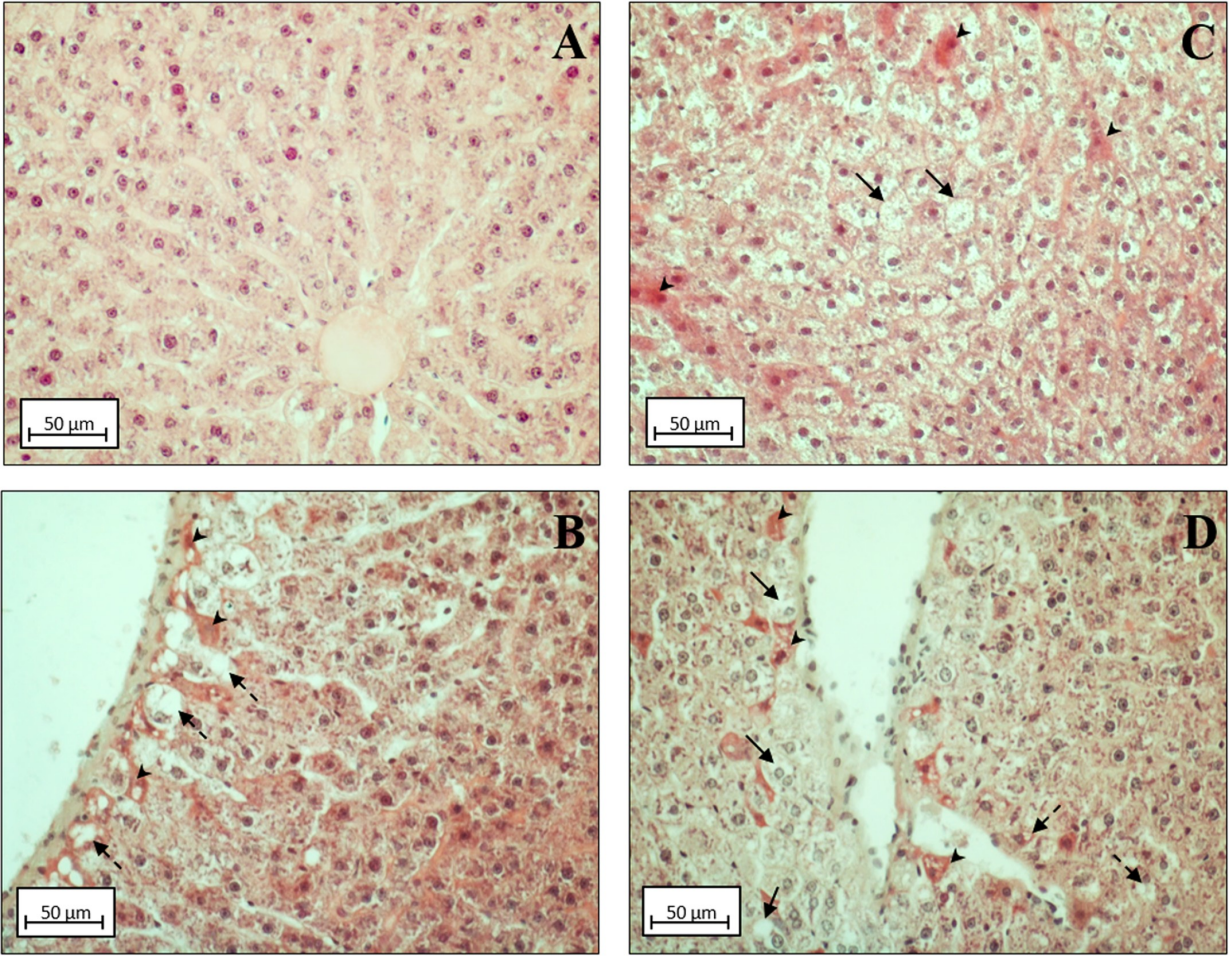
Liver histology. Sections of liver stained with Hematoxylin and Eosin. A) Control rats (N group). Lipid accumulation in HFD condition (black arrows, panel C, D group). Perivasal cellular vacuolization in presence of DDE (dashed arrows, panels B (N+DDE) and D (D+DDE). Eosinophilic cells in HFD (panel C) and in DDE-treated animals (panel B, N+DDE & panel D, D+DDE) were evidenced (arrowheads). Magnification used: 10X; Scale bar 50 μm.

**Fig 3:**
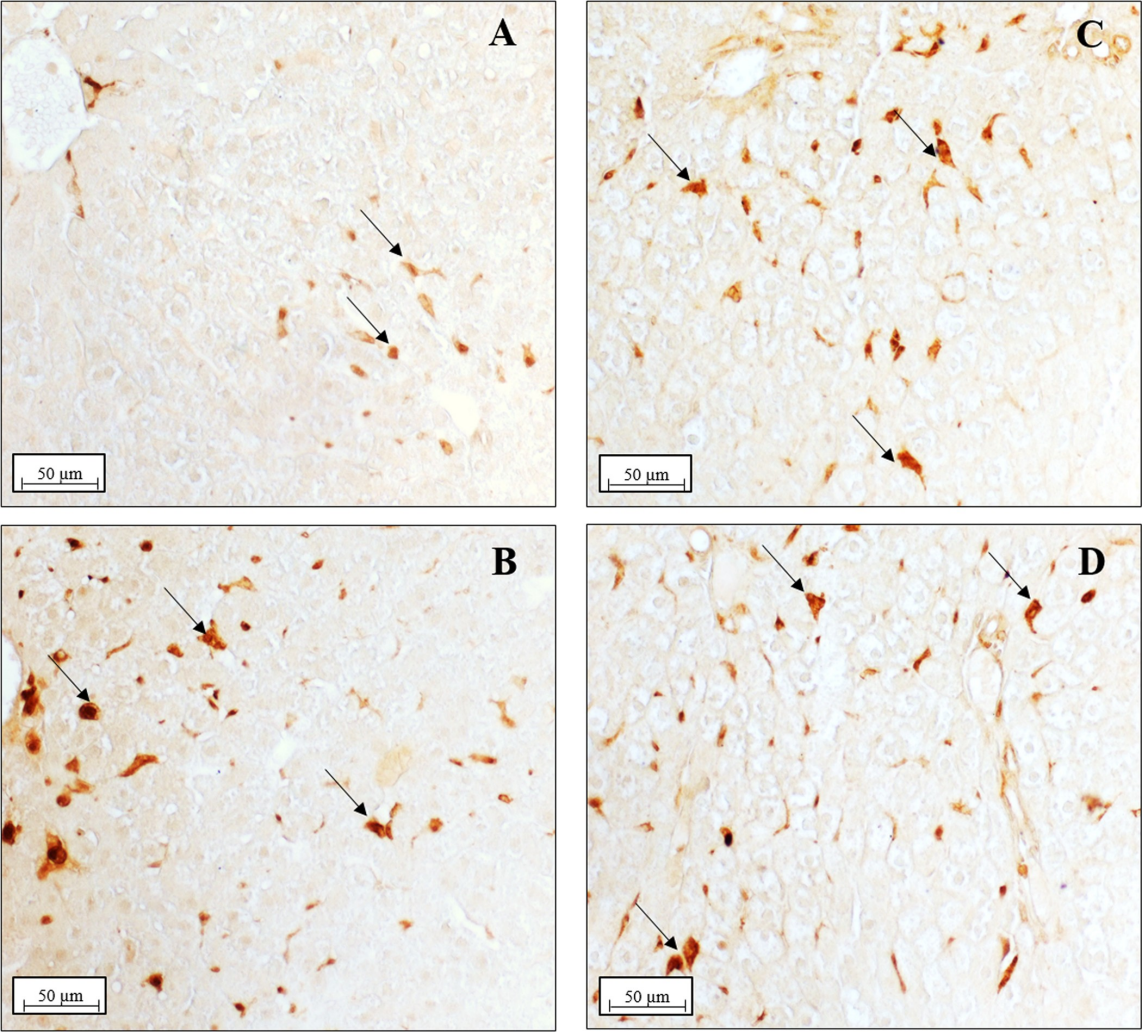
Hepatic Kupffer cells detection. Sections of liver were immunostained with anti-CD68 antibody to detect Kupffer cells in the liver. Panel A, N group; panel B, N+DDE group; panel C, D group; panel D, D+DDE group. Positive cells were evidenced with black arrows. Magnification used: 10X; scale bar applied: 50 μm.

Morphological analysis showed a variable content of lipid droplets (LD) according to the different experimental groups. The lower content of LD was present in N and N+DDE groups, (Fig 2A & 2B) while, the higher content of LD was in D group, with several ballooning hepatocytes (Fig 2C). An intermediate accumulation of lipid was evident in D+DDE group (Fig 2D). In both DDE-treated animals, many LD gave a vacuolated appearance to the cells around vessels (Fig 2B & 2D). The liver of D+DDE and N+DDE groups showed—several cells strongly eosinophilic. Moreover, anti-CD68 antibody immunostaining analysis to detect Kupffer cells in the liver showed an increased number of Kupffer cells in all treated groups vs. N (Fig 3).

### High fat diet and DDE induce oxidative stress in the liver

**Fig 4:**
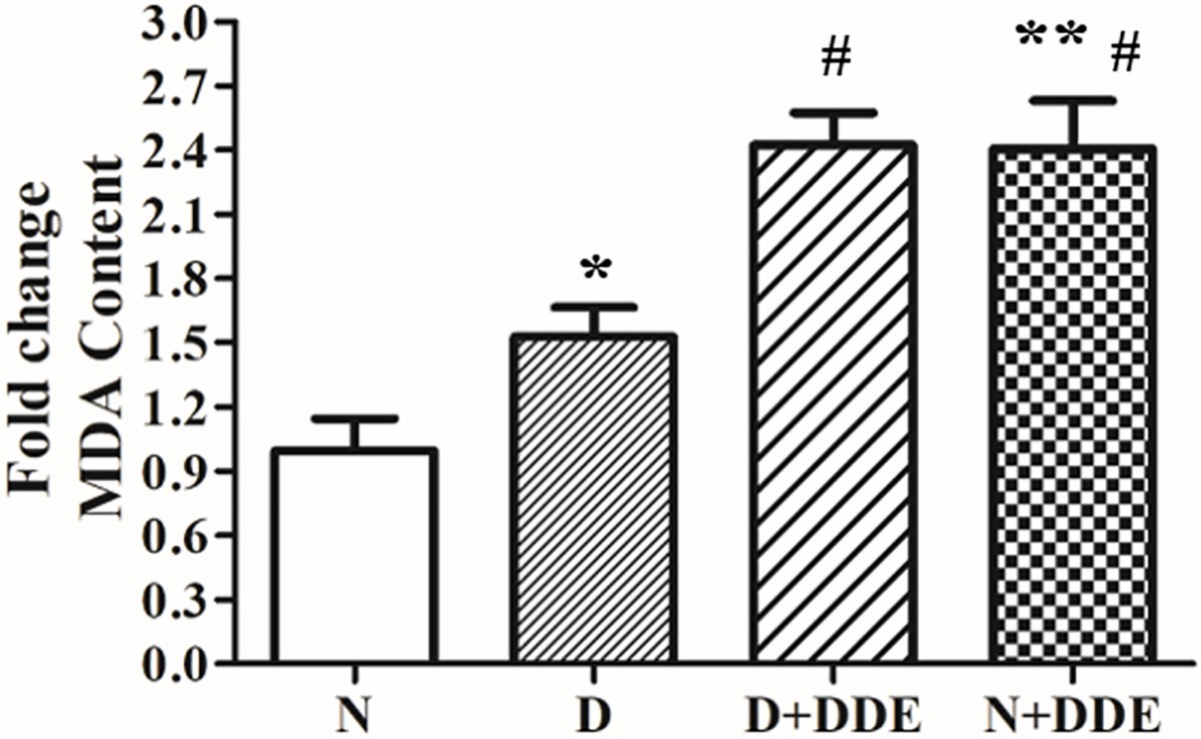
Hepatic lipid peroxidation. Analysis of the hepatic lipid peroxidation measured as MDA content in rat liver homogenate. Significance of differences is shown: *p<0.*05* vs. N; ** *p<0*.*01* vs. N; #*p<0*.*05* vs. D.

**Fig 5:**
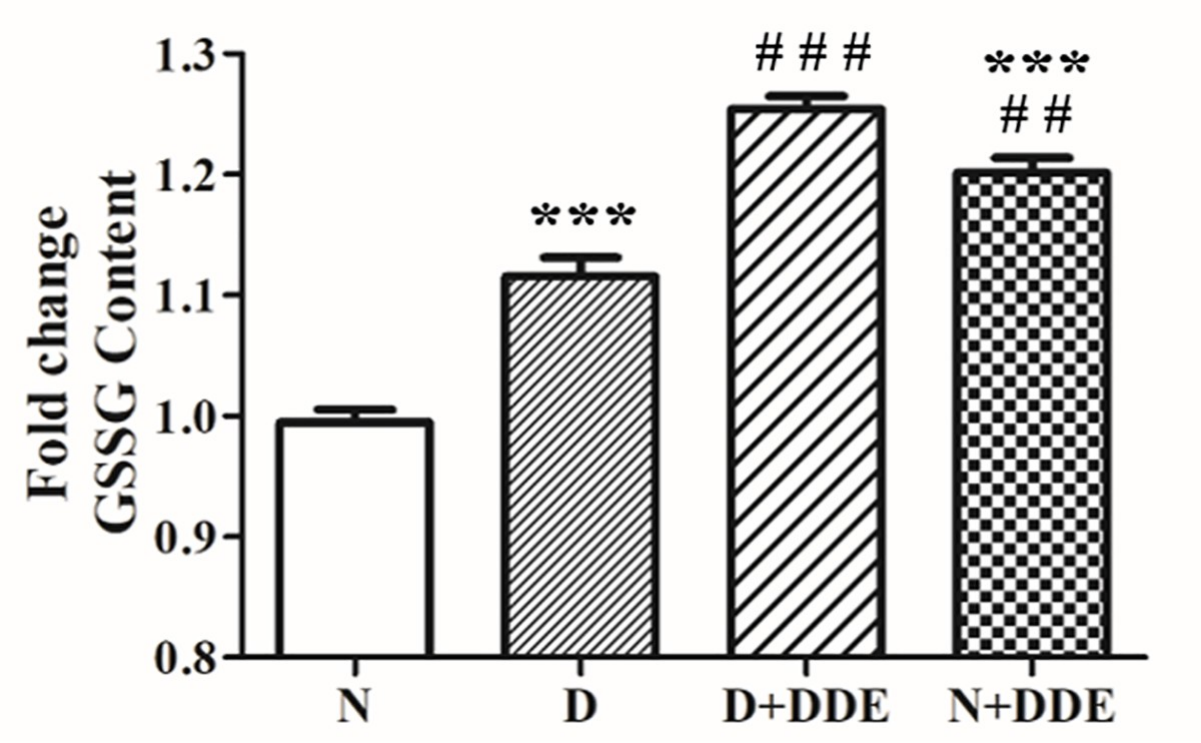
GSSG hepatic content. The level of GSSG was evaluated on total liver homogenate. Significant of differences is shown: ***p<0.*001* vs. N; *## p<0*,*01* vs. D; ### *p<0*.*001* vs. D.

Hepatic oxidative stress was confirmed monitoring the effect of the treatments on lipid peroxidation (in terms of MDA content) and GSSG levels in the total homogenate. Our results showed significant fold increase of MDA in D group vs. N (~1.5-fold), in N+DDE vs. N and vs. D (~2.4-fold and ~1.5-fold respectively) and in D+DDE vs. D (~1.6-fold). Furthermore, we did not detect significative differences of MDA accumulation between D+DDE and N+DDE groups (Fig 4). Regarding GSSG content, an important parameter to evaluate the rate of oxidative stress in the tissue, our data showed slight but significant fold increase in D group, vs. N (~1.1-fold); in D+DDE group vs. D (~1.2-fold) and in N+DDE vs. N (~1.2-fold). Statistical analysis indicated no-changing of hepatic GSSG levels between DDE-treated animals (Fig 5).

### Changing in content and activity of the main hepatic antioxidant enzymes

To gain insight into the possible mechanisms used in the liver to control, at least in part, the oxidative stress induced by treatments, we also checked changes in both cytosolic and mitochondrial superoxide dismutase (SOD1 and SOD2, respectively) and in GPx1 contents. These proteins constitute the first line of defense against cellular ROS accumulation.

**Fig 6:**
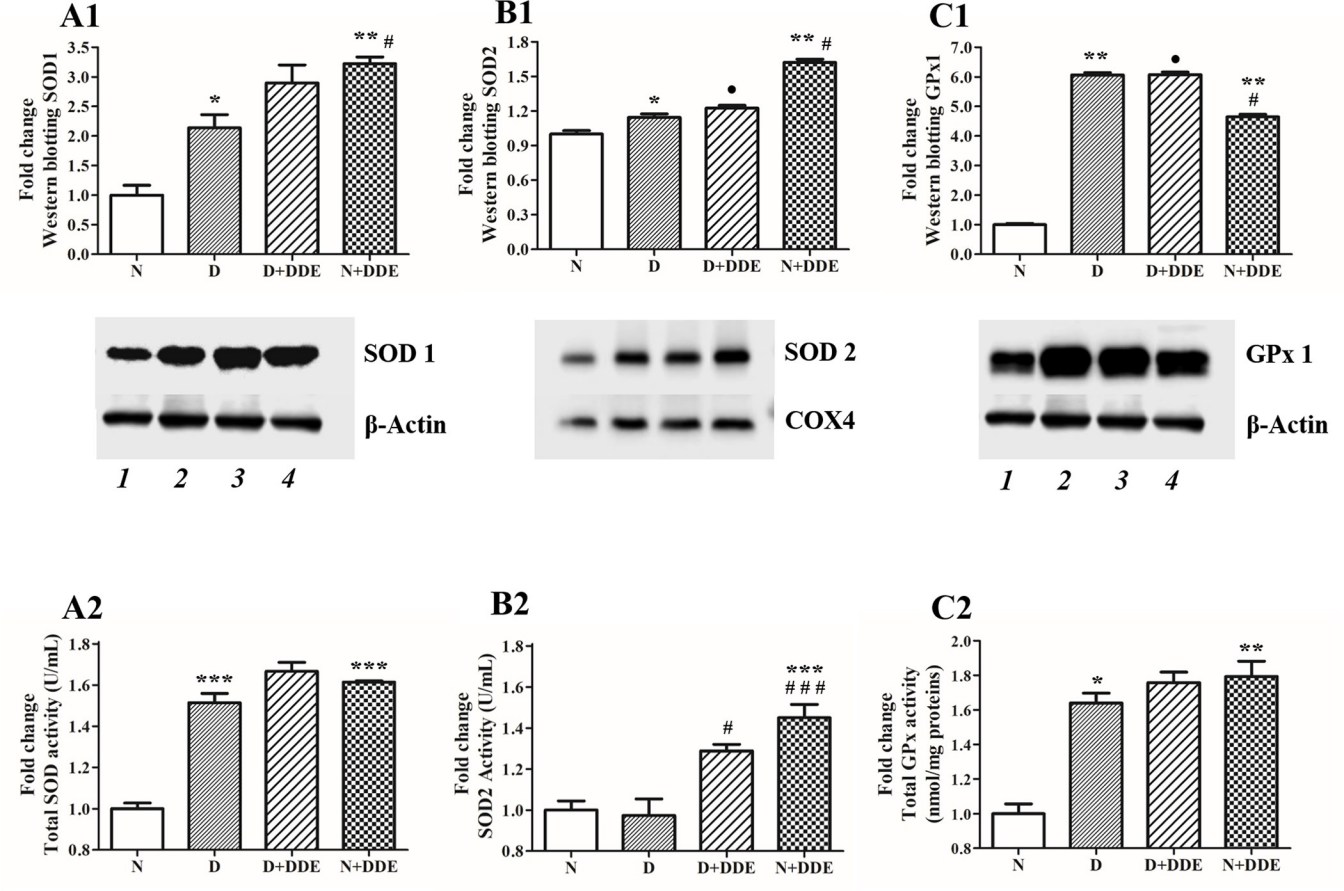
Western blotting and enzymatic activity of antioxidant enzymes. (A1) Hepatic cytosolic SOD1 content; (A2) Total SOD activity; (B1) Hepatic mitochondrial SOD2 content; (B2) SOD2 activity; (C1) Hepatic GPx1 content; (C2) Total GPx Activity. WB intensity of the bands were normalized to that of β-actin (in A1 and C1) or COX4 (in B1). Lines legend: 1, N; 2, D; 3, D+DDE; 4, N+DDE. Significance of differences is shown. *p<0.*05* vs. N; **p<0.*01* vs. N; ***p<0.*001* vs. N; #*p<0*.*05* vs. D; ##*p<0*.*01* vs. D; ###*p<0*.*001* vs. D; •*p<0*.*05* vs. N+DDE.

Regarding SODs, SOD1 protein content (Fig 6A1), showed a significant fold increase in D group vs. N (~2-fold), in N+DDE vs. N and D (~3-fold and ~1.5-fold respectively) and in D+DDE vs. D (~1.3-fold). No significative modulation of this enzyme was observed between D+DDE and N+DDE groups. Moreover, we also evaluated the total SODs activity in liver homogenates (Fig 6A2). The results indicate increased SODs activity in D group vs. N (~1.5-fold). No change in enzymatic activity was observed between D, D+DDE and N+DDE. In fact, SODs activity in DDE-treated animals was found increased similarly to D groups (~1.6-fold). For SOD2 protein content (Fig 6B1), slight but significant fold increase was observed in D vs. N (~1.2-fold). The highest fold increase was measured in N+DDE vs. N and D (~1.6-fold and ~1.4-fold respectively). No appreciable variation of the protein content was found between D+DDE and D groups. Therefore, in D+DDE group, a lower protein content was observed when compared with the N+DDE group (~0.7-fold). Regarding SOD2 activity (Fig 6B2), no significative variations were found in D vs. N, whereas increased SOD2 activity was found in D+DDE vs. D group (~1.3-fold). Moreover, according to western blotting data, the highest increase in SOD2 activity was found in N+DDE group vs. N and vs. D (~1.5-fold).

Finally, concerning GPx1 (Fig 6C1), we found significant fold increase of the protein content in all treated groups vs. N, particularly in high fat fed animals. In fact, we observed the highest GPx1 content in D vs. N (~6-fold). Significant fold increase of GPx1 was also observed in N+DDE vs. N (~4.6-fold), whereas no difference was found between D+DDE and D groups. Comparing the DDE-treated animals, GPx1 was slightly abundant in D+DDE than N+DDE (~1.3-fold). According to GPx1 protein levels, total GPx activity increased in all treated groups vs. N (~1.65-fold in D, ~1.75-fold in D+DDE and ~1.8-fold in N+DDE), without changes among treated groups (Fig 6C2).

### UCP2 gene expression and mRNA localization

**Fig 7:**
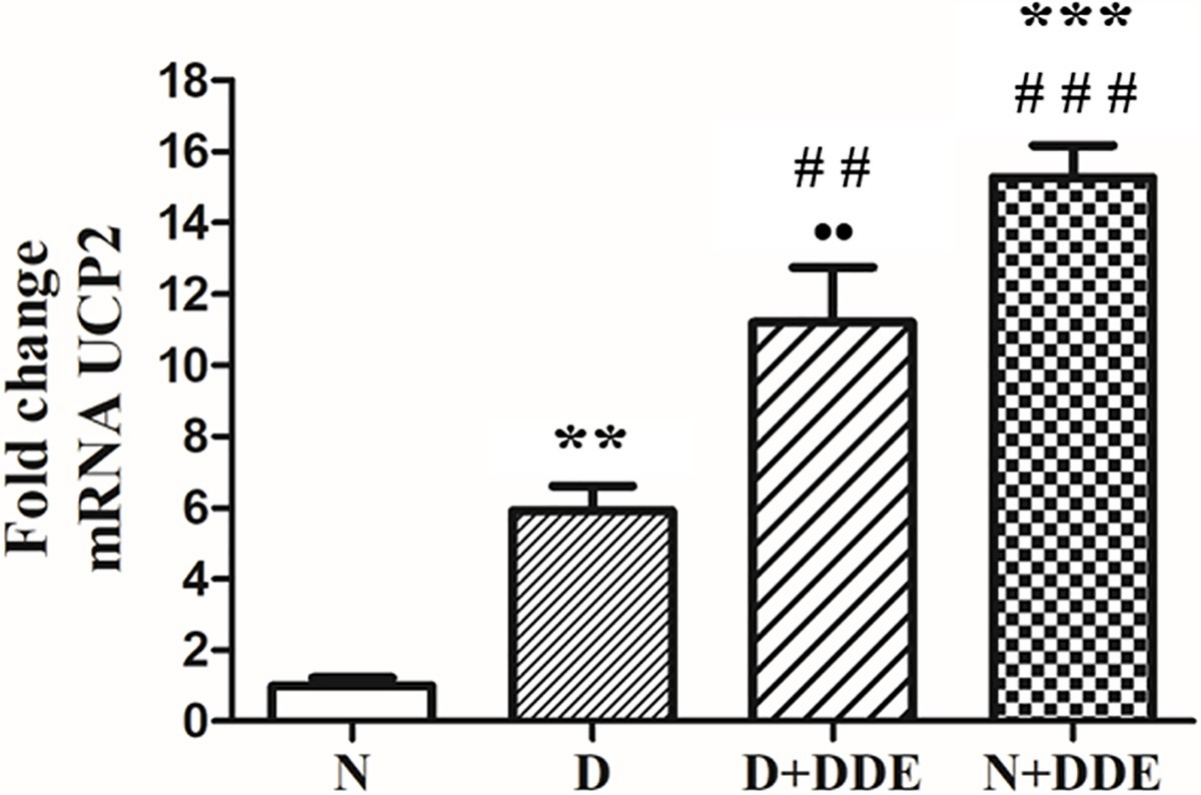
UCP2 gene expression in rat liver. UCP2 mRNA levels were determined by using real-time PCR analysis. The amount of UCP2 transcripts was normalized to that of β-actin mRNA and converted in fold change, compared with rats fed with a standard diet (N group). Significance of differences is shown. **p<0.*01* vs. N; ***p<0.*001* vs. N; ##*p<0*.*01* vs. D; ###*p<0*.*001* vs. D; ••*p<0*.*01* vs. N+DDE.

Once established the quality of the designed primers, able to amplify with a conventional PCR a single band corresponding to an UCP2 cDNA fragment of 230 bp, we investigated the relative abundance of UCP2 transcripts in the livers from the different groups of rats. We found a detectable UCP2 expression in N group, thus indicating that in liver is present a cellular population in which this mitochondrial protein is constitutively expressed (Fig 7, N group). A significant fold increase of UCP2 mRNA level was observed in D vs. N (~5.8-fold), in N+DDE vs. N and vs. D (~15-fold and 2.6-fold respectively) and in D+DDE vs. D (~2-fold); in D+DDE group, a lower content of UCP2 transcript was observed vs. N+DDE (~0.7-fold) (Fig 7, D, D+DDE and N+DDE) groups, respectively.

**Fig 8:**
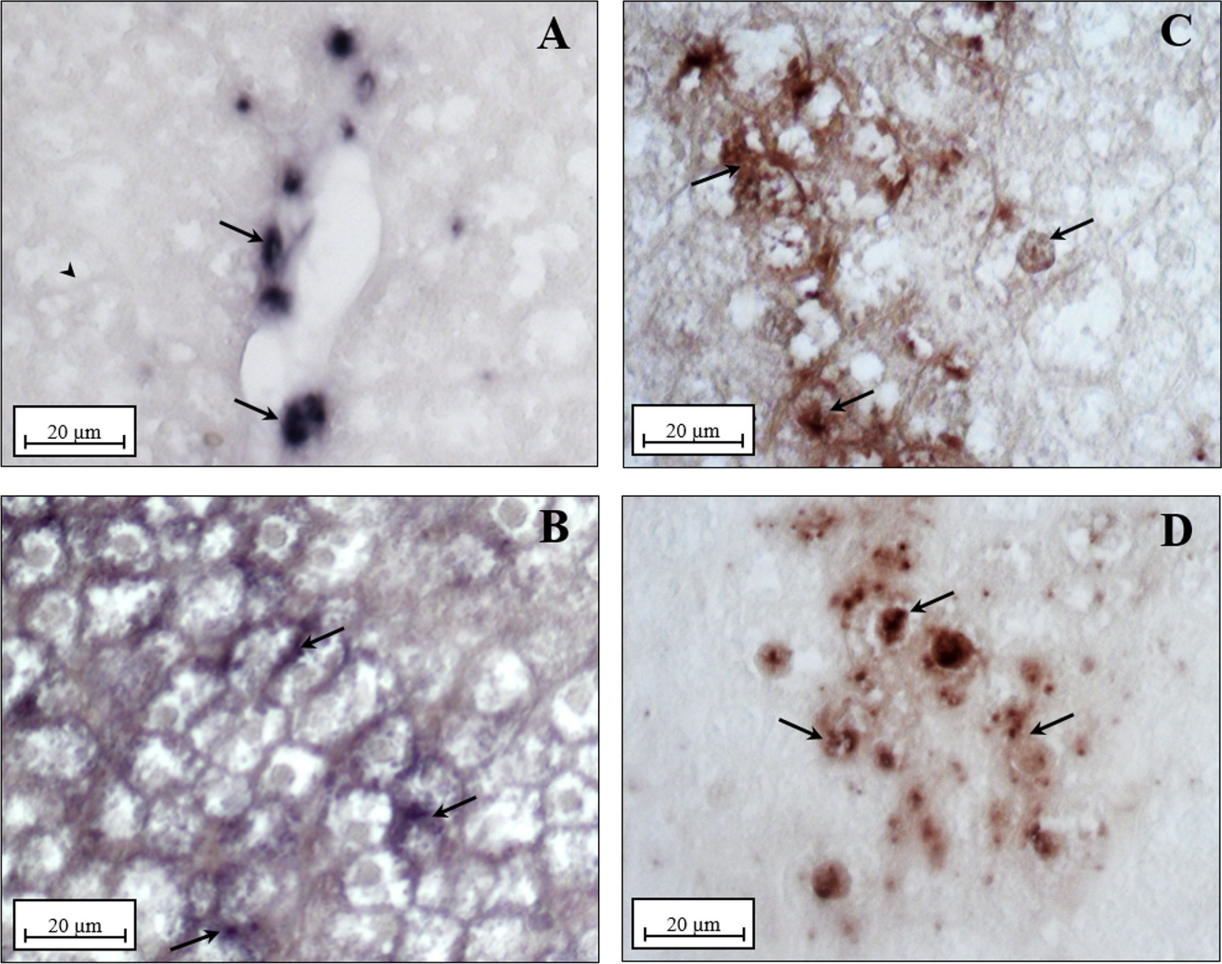
UCP2 mRNA localization in rat liver. Section of liver from rats fed with a standard diet (N, panel A): transcripts are localized only in Kupffer cells (black arrow), hepatocytes are unlabeled (arrowhead). In all the other groups (N+DDE, D, D+DDE, panels B, C and D respectively) transcripts are localized in both Kupffer cells and hepatocytes. Magnification used: 20X; Scale bar: 20 μm.

In *situ* hybridization analysis performed by using the same cDNA fragment, showedin N group the Kupffer cells as the unique site of the basal UCP2 gene expression (Fig 8A), as previously reported by Larrouy et al., (1997) [42]. On the contrary, in all the other groups (D, N+DDE, D+DDE) UCP2 transcripts were detected in both Kupffer cells and hepatocytes (Fig 8B, 8C and 8D).

### UCP2 protein detection

**Fig 9:**
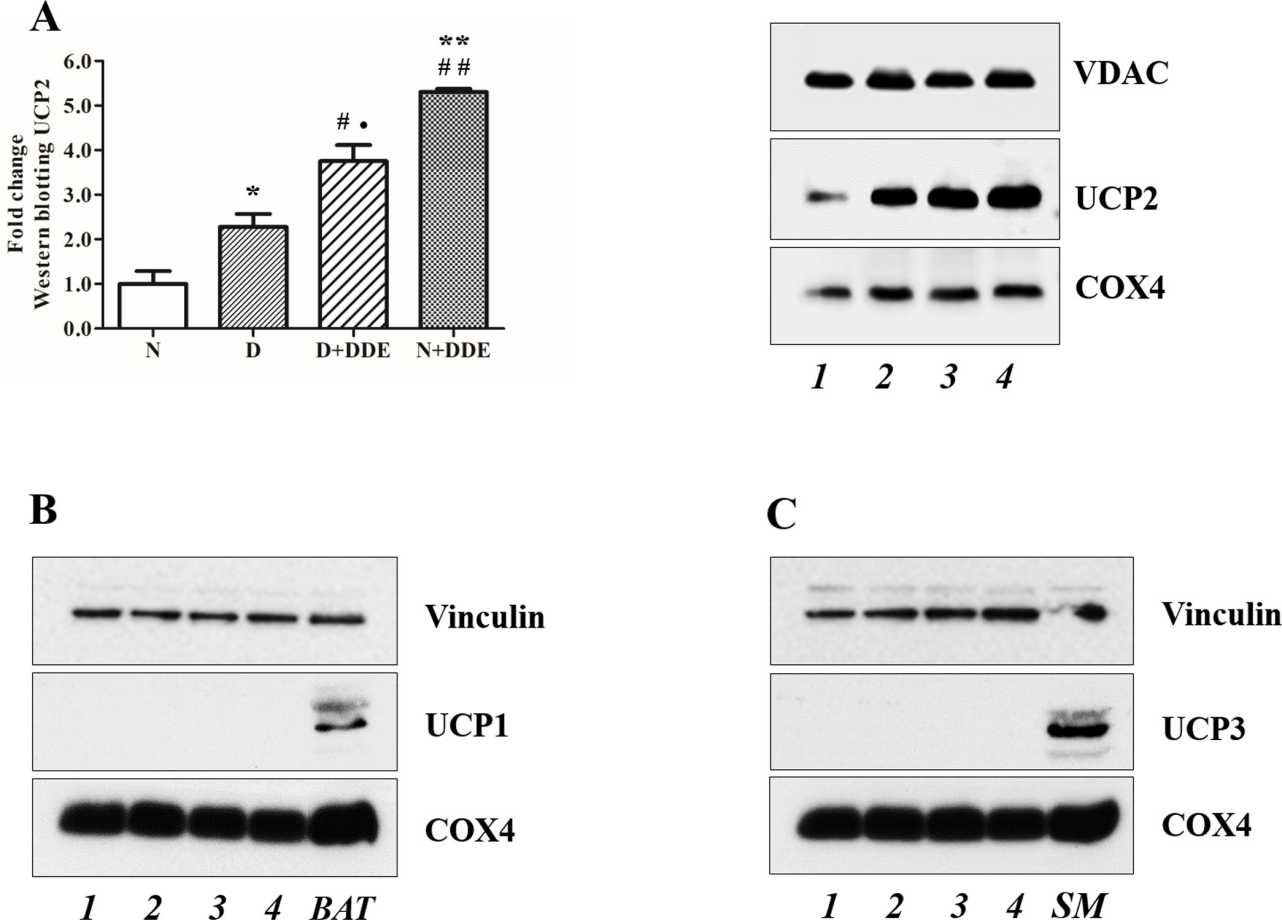
UCP2 Western blotting. (A) Western blotting for the mitochondrial protein UCP2. The amount of UCP2 protein content was normalized to COX4 and converted in fold change, compared with rats fed with a standard diet (N group). (B) Western blotting for UCP1; (C) Western blotting for UCP3. Significance of differences for UCP2 protein levels are shown: *p<0.*05* vs. N; **p<0.*01* vs. N; #*p<0*.*05* vs. D; ##*p<0*.*01* vs. D; •*p<0*.*05* vs. N+DDE. Lines legend: 1, N; 2, D; 3, D+DDE; 4, N+DDE.

**Fig 10:**
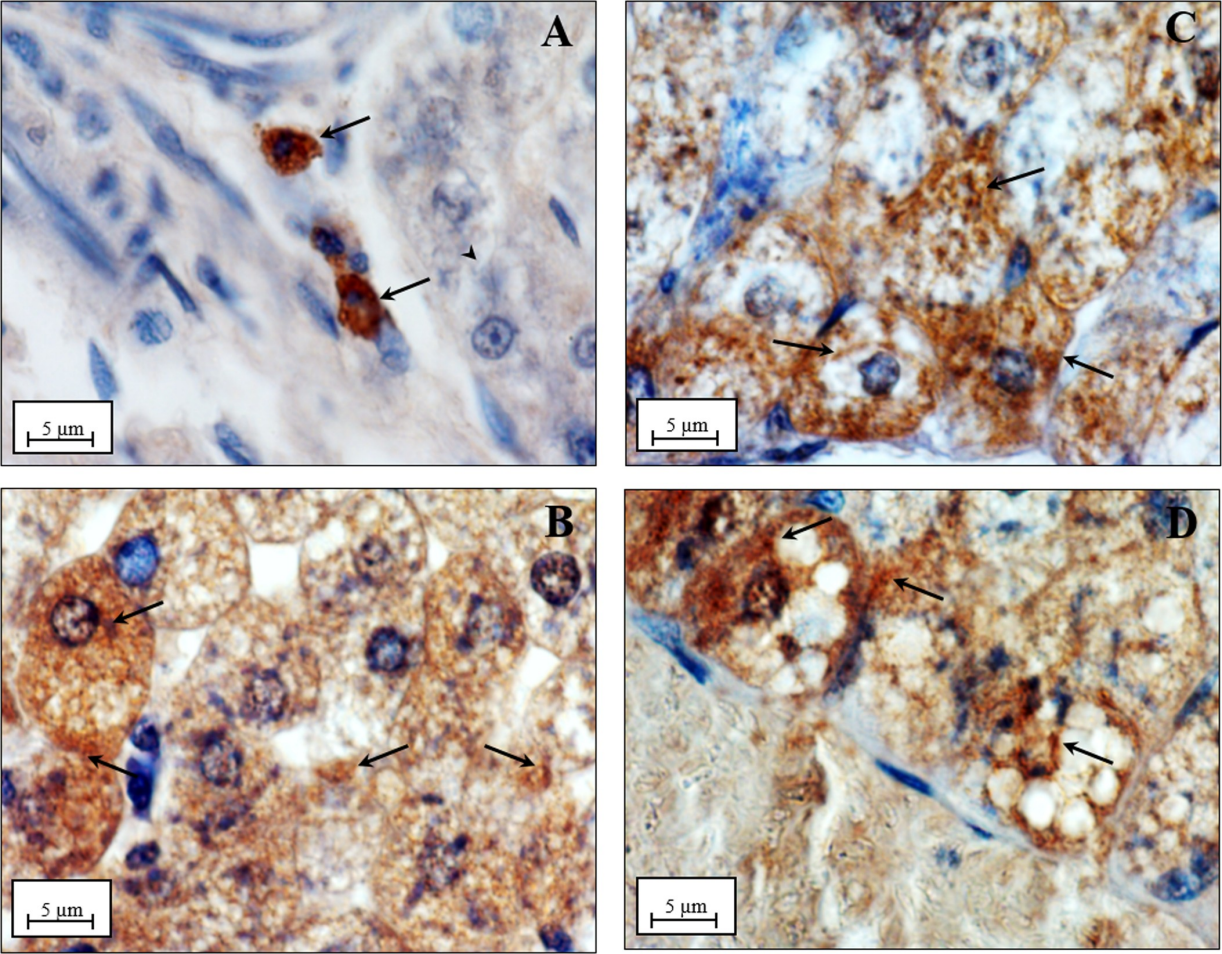
UCP2 Immunolocalization. (A) Under normal diet condition (N group), only phagocytic cells were marked with primary antibody (black arrows), whilst no positivity was detected in the hepatocytes (arrowhead). (C) Immunoreactive hepatocytes (black arrows) were evident following HFD condition and in DDE-treated groups (panel B, D group; panel D, D+DDE group). Notice mitochondria labeled by anti-UCP2 antibody, thus confirming the reliability of the immunostaining. Magnification used: 100X; Scale bar 5μm.

**Fig 11:**
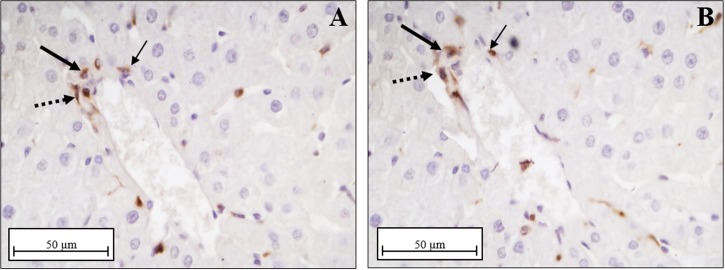
CD68 and UCP2 localization in N rats. Immunostaining for CD68 (A) and UCP2 (B) reelevates colocalization of the two antigens. Different arrows were used to compare the same cells positives to the both antibodies used (big arrow, thin arrow, dashed arrow). Magnification used: 40X; Scale bar 50μm.

Different technical approaches were used for the UCP2 detection. Firstly, western blotting analysis (Fig 9A), indicated a significant fold increase of UCP2 protein content in D vs. N (~2.3-fold), in N+DDE vs. N and vs. D (~5.3-fold and 2.3-fold respectively) and in D+DDE vs. D (~1.6-fold). In according to the UCP2 mRNA levels previously described, a lower UCP2 protein content was detected in D+DDE group vs. N+DDE (~1.4-fold). Regarding UCP1 and UCP3, western blotting analyses were done on total tissue homogenate. The results did not show protein bands in the liver but showed antibody reactivity only in the tissues used as positive control: brown adipose tissue for UCP1 and skeletal muscle for UCP3 (Fig 9B and 9C). Moreover, immunohistochemical analysis for UCP2 demonstrated a low positivity in N animals (Fig 10A), limited to the Kupffer cells. In D group, the immunostaining for UCP2 appeared in the mitochondria of hepatocytes (Fig 10C) and increased sharply in mitochondria of DDE-treated groups (Fig 10B–10D). To confirm that, in N rats, UCP2 localizes principally in the Kupffer cells, adjacent sections on the slide were immunostained with CD68 (marker of macrophages, Fig 11A) and UCP2 antibody (Fig 11B). Data showed that same cells are positives to both antibodies analyzed.

### DDE stimulates cytochrome P450 2B

**Fig 12:**
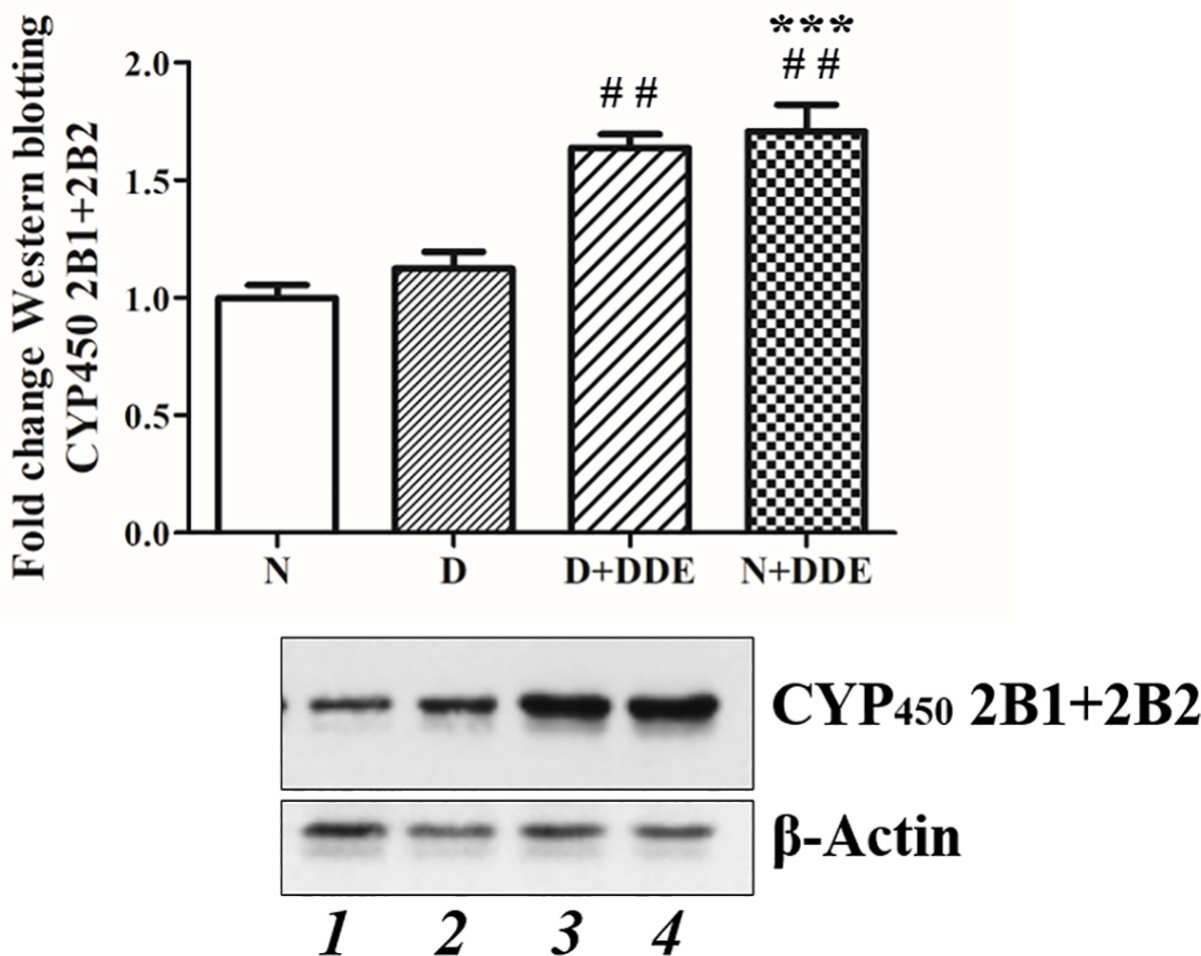
CYP450 2B protein levels. Western blotting analysis of CYP450 2B. Data were obtained as media ± standard deviation and graphically represented as fold change vs. N. Significant of difference between groups is shown: ***p<0.*001* vs. N; ##*p<0*.*01* vs. D.

Western blotting analyses showed induction of cytochrome P450 2B (CYP2B) protein levels in N+DDE vs. N (1.70-fold) and vs. D (1.50-fold) and in D+DDE vs. D (1.45-fold). No significative differences were found between DDE-treated animal groups (Fig 12).

## Discussion

This work was carried out to study the cellular response, if any, to the toxic actions of the pesticide DDE in rat liver following a treatment of four weeks, in which DDE was administered alone or in combination with hyperlipidic diet to compare the effects of single exposure (xenobiotic or HFD) and simultaneous exposure to both environmental stimuli. Our findings revealed the presence of a different degree of oxidative stress for the different experimental groups used, together with the modulation of gene expression and protein synthesis of UCP2, the uncoupling protein that seemed to be involved in the regulation of oxidative damage as adaptive cellular response, so supporting the endogenous antioxidant system of hepatocytes. Moreover, our research confirmed that UCP2 in the hepatocytes under oxidative stress conditions was mainly induced by DDE.

Starting from analysis of obesity development and lipid profile, body weight gain and visceral WAT pad weight as well as serum triglycerides and cholesterol levels, and hepatic lipid content were found significantly increased only in HFD-treated animals confirming that high fat diet induces obesity and alters serum lipid profile and fatty acids deposition in the organs. Particularly for triglycerides, in D rats we found the highest serum triglycerides and cholesterol levels compared to the other groups. These data agree with our previous results obtained in rats, that showed altered serum lipid profile in HFD-feed animals after a treatment period of 6 weeks [5]. Moreover, mitochondrial fatty acids β-oxidation and CPT system activity were found increased in D vs. N confirming previous finding [5] and suggesting that the increased capacity of mitochondria to oxidize fatty acids in HFD fed rats might serve as a compensatory mechanism for the elevated hepatic fatty acids uptake that occurs during high fat feeding [5]. However, the increased fatty acid oxidation was not able to completely counteract the increased load of hepatic free fatty acids, resulting in hepatic lipid accumulation in D group, as confirmed by hepatic lipid content and histological analysis. Noteworthy, the increased mitochondrial fatty acid oxidation might play a crucial role in enhancing ROS production [56]. In accordance, D group showed an increased content of MDA, a product of lipid peroxidation acting as a marker of oxidative stress. Increased oxidative stress may in turn lead to activation of endogenous antioxidant defense system. Indeed, the SODs protein levels showed that both isoforms (cytosolic and mitochondrial) were increased in D group. As regard SODs activity, total SODs activity increased in D group, whereas no significant difference was found in SOD2 activity suggesting that the increased in total activity may be due to the other SOD isoforms. SODs up-regulation could indirectly suggest that this enzymatic system serve to control the oxidative damage produced by the lipid surplus. It is known that saturated fatty acids lead to an increase of ROS and oxidative stress in terms of superoxide anion, H_2_O_2_ and hydroxyl radicals [57]. Furthermore, the increased levels of SODs ensured the conversion of a large part of the superoxide anion in H_2_O_2_ that can be eliminated through peroxidase enzymes. In accordance, GPx1 protein expression was higher in D groups than in the other two groups, presumably elicited to the excessive hydrogen peroxide accumulation and fatty acid hydroperoxides. Total GPx activity also increased in D animals, confirming the data also reported in our previous work [58]. In addition, UCP2 increase on mitochondrial inner membrane providing a further mechanism to regulate ROS production at mitochondrial level. However, the summary of these anti-oxidant effects did not completely counteract stress induced by lipid oversupply and hepatic cell damage occurred as suggested by increased ALT and AST serum levels.

As concern the DDE-treated groups, hepatic detoxification pathways were stimulated by DDE, as suggested by the increase in CYP450 2B content. Indeed, it is known from literature data that DDE can interacts with the constitutive androstane receptor (CAR) and pregnane X receptor (PXR), activating CYP450 genes family stimulating CYP450 2B and 3A [59]. We also found a significant increase in mitochondrial fatty acid oxidation and CPT system activity in both DDE-treated groups. This increase was higher than the one found in D group, suggesting that it could be useful to support energy requirements for detoxification processes induced by DDE [59]. In D+DDE group, the increase in fatty acid utilization was associated with a lower hepatic lipid content and serum triglycerides level vs. D group. Beside this, the analysis of ALT and AST described a similar entity of liver damage in D and DDE-treated groups, where serum transaminase levels were found equally increased. Histological liver analysis confirmed hepatic alteration in all treated groups, but it showed features of hepatic steatosis and macrophages infiltration worsened in DDE-treated rats, as confirmed by CD68 immunostaining. According to the histological changes, the levels of MDA was increased in all the experimental groups compared to the control, indicating the pro-oxidant role of both saturated fatty acids and DDE in our experimental conditions, but DDE-treated groups showed the highest content of MDA suggesting a more severe DDE induced oxidative stress. The increase in both mitochondrial fatty acid oxidation and CYP2B reaction cycle in DDE-treated groups, contributed to elevate ROS production that in part was controlled by hepatic antioxidant activity. The levels of the main antioxidant enzymatic systems were also raised in all the treated groups, with some differences, sometimes apparently contradictory, among the groups.

Examining the antioxidant enzymatic systems, we observed the same protein levels of SOD1 in DDE-treated groups as well as total SOD activity, whereas, particularly for SOD2, the protein levels and the enzymatic activity depended on the type of diet to which the pesticide was associated and/or on the subcellular compartment considered. Indeed, the N+DDE group showed the highest levels of SOD2 protein levels among the treated groups. On the contrary, in the D+DDE group, SOD2 protein levels were the same of group D, i.e. significantly lower than N+DDE, but their activity no change significantly vs. N+DDE. Finally, the protein levels of GPx1 found in liver of N+DDE rats was intermediate between N and both D and D+DDE groups, while, total GPx activity was the same of HFD-treated rats. The different responses detected in the two DDE-treated groups might be arisen by different causes, the first was that the pesticide could act variously depending on the diet which it is associated, as stated above. Being DDE a hydrophobic molecule, it tends to be retained in the lipid matrix of adipose tissue or in the lipid droplets observed in the hepatocytes, probably in a partially inert form. In the D+DDE group, total SODs and GPxs protein activities were the same measured in D group, thus suggesting that the cellular responses of the antioxidant system in this group are generated by the increased amount of fatty acids, rather than DDE presence. These findings allow us to hypothesize that the most of DDE administered together with the hyperlipidic diet had been trapped inside fat, in line with the found increases in both WAT weight and hepatic ectopic lipid accumulation. Conversely, when the pesticide is administered combined with a standard diet, the anti-oxidant responses recorded in the liver cells came from properly by the pesticide. The results also demonstrate that there was no synergistic effects of fatty acids and DDE on antioxidant enzymes synthesis and activity. Indeed, the analysis on the total GSSG accumulated in the liver reelevates that D rats accumulate GSSG compared to controls, whereas DDE-treated animals exhibited a similar further increase in GSSG content vs. N and D group. The data correlates with the levels of hepatic MDA suggesting that DDE may play a predominant pro-oxidative role when it is not associated to fats.

As already described, the highest expression and activity of the mitochondrial SOD2 were detected in the hepatocytes of N+DDE rats. SOD2 is a critical protein that acts against superoxide produced by mitochondrial respiration. Homozygous SOD2 knockout mice faced to early postnatal death [60]. Heterozygous SOD2 knockout mice exhibited numerous alterations in mitochondrial function [61] and ultrastructural abnormalities (mitochondrial swelling), increased susceptibility for induction of permeability transition [62] and enhanced lipid peroxidation [63]. Instead, SOD2 overexpression protects mitochondrial respiratory function [64] and attenuates mitochondrial ROS generation, intracellular lipid peroxidation and cell death [65]. SOD2 is located within the mitochondrial matrix, in the site of free radical production from the electron transport chain (ETC). The highest SOD2 expression in N+DDE animals suggested that oxidative stress was at first generated in mitochondria and the highest superoxide production was due to DDE. Hence, this pesticide was reported to damage some components of ETC. In addition, it has been demonstrated that an impairment of ETC gives rise to increased levels of mitochondrial ROS [66]. The excess of superoxide could be controlled by ensuring that a significant proportion of protons may bypass the ATP synthase pathway and leak back to the mitochondrial matrix, namely by a mitochondrial uncoupling [67]. UCPs supported this function, representing the first line of antioxidant defense aimed at resolving mismatched outward and inward proton fluxes [68,69]. For this reason, our attention focused on UCP2 and our findings showed that UCP2 in liver of control rats was expressed only in immunocompetent cells, while the oxidative stress, produced by the different treatments, induced mRNA synthesis and protein translation in hepatocytes too. These data agree with those of Chavin and coworkers [43], who found UCP2 upregulation in hepatocytes of obese rats. The levels of mRNA and protein increased in all the treated groups, but the highest level was recorded in N+DDE group, confirming our hypothesis of the highest pesticide pro-oxidative activity in association with the normal diet regimen. According to Echtay et al., (2002) [70] and Brand et al. (2004) [71], UCP2-mediated proton leak requires activation by superoxide and lipid peroxidation derivatives such as 4-hydroxynonenal and other reactive alkenals. In N+DDE, as described above, the highest SOD2 and UCP2 expression was found, accompanied with elevated levels of MDA. We assumed that SOD2 and UCP2 co-operate to limit the production of mitochondrial superoxide anion in response to oxidative damage induced by the pesticide. Therefore, the similar MDA levels measured in the two DDE treated experimental groups, can be explained according to a different hypothesis. In particular, in N+DDE group mitochondrial ROS production is limited by the high expression of SOD2 and UCP2, while in D+DDE, the major content of fat could cause a minor activity of the pesticide, but it could supply a great amount of lipid to peroxidation process. In fact, this situation generates cellular responses partly regulated by saturated fats.

From our findings we conclude that, in our experimental conditions, both saturated fatty acids and DDE induce oxidative stress in the liver. Hepatocytes activate the endogenous antioxidant system to protect themselves from the oxidative damage predominantly generated in DDE-treated animals. In this system, the induction of UCP2 in the hepatocytes can be used as adaptive cellular response to limit the damage produced by an excessive mitochondrial superoxide generation, mainly caused by DDE. Therefore, UCP2 may have a primary role as a sensor and suppressor of mitochondrial ROS, with increasing expression and functions at increasing levels of oxidative stress [72]. With the limitation that further studies are needed to gain insight in the mechanisms involved in DDE effect, the results of the present study suggest that hepatic oxidative stress is induced by both HFD and xenobiotics exposure, but the anti-oxidant defense responses are differently modulated, with a possible role of UCP2 mainly in the response to xenobiotics.
